# Successful closure of a detached muscle layer during gastric endoscopic submucosal dissection with fibrosis using clips with the use of polyglycolic acid sheets and fibrin glue

**DOI:** 10.1055/a-2257-3687

**Published:** 2024-02-22

**Authors:** Satoshi Abiko, Koji Hirata, Kazuharu Suzuki, Kenji Kinoshita, Kazuteru Hatanaka, Yoshiya Yamamoto, Hirohito Naruse

**Affiliations:** 1 Department of Gastroenterology and Hepatology, Hakodate Municipal Hospital, Hakodate, Japan

Resections of gastric tumors with severe fibrosis by the endoscopic submucosal dissection (ESD) procedure are still difficult in some cases 1 . Perforation is a major complication of ESD with severe fibrosis 2 ; however, there has been no report of adverse events such as a huge, detached muscle layer. Here we report successful closure of a huge, detached muscle layer during gastric ESD with severe fibrosis using clips with the use of polyglycolic acid (PGA) sheets and fibrin glue.

ESD was performed for a 74-year-old man with gastric adenocarcinoma on an ulcer scar in the lesser curvature of the angle of the stomach. During ESD, we observed very strong fibrosis and carefully performed submucosal dissection. We had unknowingly removed both the lesion and some of the muscle layer. After resection of the lesion, there was a huge, detached muscle layer of about 30 mm and we observed the lesser omentum ( Fig. 1 **a** ). The site was closed using clips ( Fig. 1 **b** ). To further protect the ulcer base, it was covered with several small PGA sheets (2×1 cm) using the method proposed by Takimoto et al. 3 . Finally, fibrin glue was sprayed ( Video 1 **,** Fig. 1 **c** ). Fortunately, computed tomography after the procedure revealed no perforation. The ulcer was on a healing trend after 6 days ( Fig. 2 **a** ). The length of the hospital stay was 15 days. After about 4 months, the ulcer had completely healed ( Fig. 2 **b** ). Careful inquiry into the patientʼs medical history revealed that he had experienced a perforation of a gastric ulcer and had undergone conservative treatment.

**Fig. 1:**
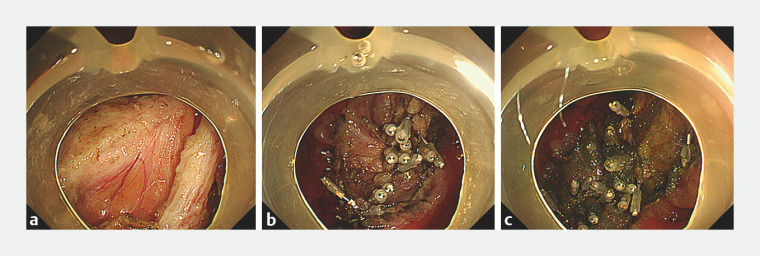
Condition after endoscopic submucosal dissection (ESD). **a** After resection of the lesion, there was a huge, detached muscle layer of about 30 mm and we observed the lesser omentum. **b** The huge, detached muscle layer during ESD was closed using clips. **c** To further protect the ulcer base, the ulcer base was covered with several small PGA sheets (2×1 cm) using the method proposed by Takimoto et al.

Video shows closure of a huge, detached muscle layer during gastric endoscopic submucosal dissection (ESD) with severe fibrosis using clips with the use of polyglycolic acid sheets and fibrin glue.Video 1

**Fig. 2:**
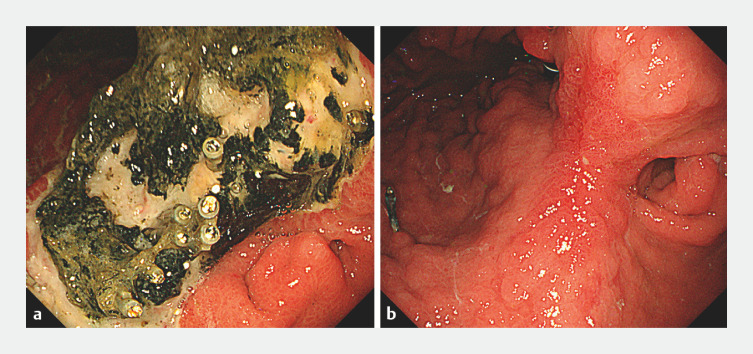
Endoscopic imaging of the clinical course. **a** The ulcer was on a healing trend after 6 days. **b** After about 4 months, the ulcer had completely healed.

When performing ESD on the stomach with severe fibrosis, it may be advisable to keep in mind the possibility of adverse events such as a detached muscle layer.

Endoscopy_UCTN_Code_TTT_1AO_2AG

## Notes

Endoscopy E-Videos https://eref.thieme.de/e-videos
*E-Videos* is an open access online section of the journal *Endoscopy* , reporting on interesting cases and new techniques in gastroenterological endoscopy. All papers include a high-quality video and are published with a Creative Commons CC-BY license. Endoscopy E-Videos qualify for HINARI discounts and waivers and eligibility is automatically checked during the submission process. We grant 100% waivers to articles whose corresponding authors are based in Group A countries and 50% waivers to those who are based in Group B countries as classified by Research4Life (see: https://www.research4life.org/access/eligibility/ ). This section has its own submission website at https://mc.manuscriptcentral.com/e-videos .
